# Discrimination against and Associated Stigma Experienced by Transgender Women with Intersectional Identities in Thailand

**DOI:** 10.3390/ijerph192416532

**Published:** 2022-12-09

**Authors:** Pimwarat Srikummoon, Yuphayong Thanutan, Natthaporn Manojai, Sukon Prasitwattanaseree, Nachale Boonyapisomparn, Unyamanee Kummaraka, Chanapat Pateekhum, Phisanu Chiawkhun, Chayut Owatsakul, Benchalak Maneeton, Narong Maneeton, Suttipong Kawilapat, Patrinee Traisathit

**Affiliations:** 1 Department of Statistics, Faculty of Science, Chiang Mai University, Chiang Mai 50200, Thailand; 2 Data Science Research Center, Department of Statistics, Faculty of Science, Chiang Mai University, Chiang Mai 50200, Thailand; 3 Mplus Foundation, Chiang Mai 50100, Thailand; 4 The Foundation of Transgender Alliance for Human Rights, Bangkok 10160, Thailand; 5 Department of Psychiatry, Faculty of Medicine, Chiang Mai University, Chiang Mai 50200, Thailand; 6 Research Center in Bioresources for Agriculture, Industry and Medicine, Department of Statistics, Faculty of Science, Chiang Mai University, Chiang Mai 50200, Thailand

**Keywords:** discrimination, transgender women, ethnic minority, monk, Thailand

## Abstract

Although Thailand is overtly open to diversity and promotes equality, discrimination of minorities based on gender, ethnicity, and/or certain occupations is unfortunately still prevalent. Society either obstructs their inclusion or accepts them but only under certain conditions. The objective of this study is to examine the discrimination of TGWs with intersectional identities within Thai society. A total of 19 TGW participants were recruited and underwent in-depth thematic interviews about their experiences of discrimination. Rechecking of the extracted information from the interview transcripts and the subsequent encoding process were conducted using the NVivo program. The results show that the median age was 30 years old, and the majority of the individuals with intersectional identities were ethnic minority TGWs (47%). The in-depth interviews were divided into four main themes, including discrimination at an educational institution, discrimination in the workplace, discrimination in daily life, and discrimination at a healthcare facility. Our findings reflect problems associated with multiple sources of discrimination aimed at transgender women with an intersectional identity in Thailand in every aspect, including harsh speech or physical abuse; occupational, social, and legal inequality; and healthcare provision disparity. Raising awareness about gender diversity and intersectionality, as well as enforcing anti-bullying legislation and anti-discrimination laws, should be continually pursued in order to protect the rights and improve the quality of life of transgender individuals with an intersectional identity.

## 1. Introduction

The lesbian, gay, bisexual, transgender, queer (LGBTQ) community is a marginalized group of people who face much discrimination around the world [1]. Although Thai society has become more open to most forms of divergence and diversity, and LGBTQ self-expression at educational institutions, in the workplace, and at healthcare facilities is tolerated, inequalities and discrimination toward them are still prevalent [2]. Discrimination is frequently directed at those who live their lives in a way that is different from the majority, such as being LGBTQI [3], from a marginalized ethnic group [4], or a sex worker [5]. Being discriminated against can lead to not seeking help for health problems because of the fear of humiliation and unfair treatment when visiting healthcare facilities [6,7,8,9,10].

The interconnectedness of several social characteristics, such as race, gender identity, and class, is known as intersectional identity and is regarded as a contributing factor to inequalities and interrelated systems of discrimination [11,12,13]. Intersectionality comprises many social statuses at once since people do not experience discrimination based on an independent social status, such as transgender women (TGWs) who are also a member of an ethnic minority [14]. Identifying individuals with an intersectional identity will enable us to comprehend them in the social contexts of family, school, workplace, and community.

Discrimination and experiences of mistreatment at educational institutions can be particularly challenging for LGBTQ students; their experiences include hate speech and sexual harassment from teachers and other students [15,16,17,18]. Sixty-one percent of TGWs were discriminated against when they were students (the highest among LGBTQ people [1]), and because this is a sensitive period for most individuals, bullying and victimization due to their self-expression and behavior caused the most pain. People from marginalized ethnic groups are more likely to be undereducated, be stateless, not have the correct documentation, and be unable to find steady high-paying employment [4], which can make their lives difficult.

TGWs frequently face discrimination concerning employment, such as limited access to job opportunities, being turned down by potential employers, being laid off, and being denied career training or promotion opportunities [2]. From a survey on the economic inclusion of LGBTQI groups in Thailand by the World Bank in 2018 [19], 77% of transgender people were denied job applications due to their gender identification, while 40% said they had experienced harassment, bullying, and/or discrimination at work. From a survey of LGBTQI people in the US in 2021 [20], 45.5% of LGBTQI workers experienced unfair treatment at work, including being fired, not hired, or harassed because of their sexual orientation and/or gender identity at some point in their lives. Interestingly, 8.9% of employed LGBTQI people reported that they had been fired or not hired because of their sexual orientation or gender identity in the past year. Of these, 11.3% were colored and 6.5% were white, thus reflecting discrimination based on their intersectional identity due to race. From the interviews concerning discrimination against TGWs who have become sex workers in Thailand, it appears that although the stigma of being involved in the sex industry is prevalent in Thai society, the high compensation and ability to express their gender identity have encouraged some of them to do so [21]. However, the downsides are no access to basic social security, no legal support, and being shunned by Thai society [5]. In contrast, the findings from a previous study in Thailand infer that LGBTQI people with a higher socio-economic status experienced less discrimination and were more unlikely to experience discriminatory encounters [22].

A previous study by Riggs et al. (2018) revealed that transgender and nonbinary people living in Australia and the UK were more likely to be subjected to family violence than other individuals [23]. In Thailand, a survey among LGBTQI people in 2019 indicated that 56.4% of TGWs suffered more discrimination from their families (53.2%) compared to other gender minorities (50.0%, 49.8%, and less than 45.0% for non-binary, bisexual men, intersex, and other groups, respectively) [1]. Moreover, they are often forced to dress contrary to their gender identity and to change their appearance to that of a man by family members (often violently) and are also bullied by people in their community. The majority of transgender individuals have suffered victimization due to their gender identification, including verbal harassment, being stalked, and harassment by strangers on the street [24]. Moreover, TGWs are still unable to change the title of their gender identity on government documents, visas, and passports, which has created a barrier to their rights to healthcare, education, work, freedom of movement, and non-discrimination [25].

Some Thai Buddhists believe that homosexual people are unable to control their sexual impulses and tendencies [26]. Nevertheless, in 2010, several news reports about the inappropriate behavior of novices and monks, such as monks having sex with novices and monks and novices making themselves look effeminate, wearing makeup, and wearing their robes tightly or in a way that suggested that they had breasts were published. Accordingly, some Thai talking heads discussed the inappropriateness of this behavior and that it is indicative of a morally degenerate Buddhist Sangha [26,27,28]. The sight of effeminate monks reinforces the idea that monasticism is the purview of “real men” only, and so TGWs who have undergone gender affirmation surgery are not allowed to enter the monkhood. Nevertheless, those who have not can be ordained if they have “good morality” and their appearance is not overtly different from a cisgender monk [27,29]. Many parents do not accept their sons’ sexual orientation or gender identity and force them into monkhood in the hope that the monastic life will cure them of their immoral sexual tendencies [26]. In addition, some TGWs have felt obliged to become monks since childhood due to the economic status of their family and/or their ethnicity. However, TGWs who become monks are usually discriminated against by their families and communities due to their chosen gender identity.

TGW patients are usually stereotyped and subjected to service discrimination, procrastination [30,31], profanity, ignorance [32], and/or even negative reactions from other patients when seeking medical care [33]. Furthermore, many researchers have found that people in minorities with multiple gender identities had more negative experiences than those with a single one [13,34,35]. What is even worse, people with multiple gender identities lack access to healthcare information and knowledge about fees [5], which affects their medical treatment outcomes [32].

The Mplus Foundation, Chiang Mai, and the SISTERS Foundation, Chonburi, are organizations that specialize in the prevention of the spread of human immunodeficiency virus (HIV), which can lead to acquired immunodeficiency syndrome (AIDS); other sexually transmitted diseases (STDs); and sexual health, as well as advocating LGBTQ rights in Thailand. In cooperation with both foundations, a study was conducted on the issues of discrimination against diverse identities. The study is categorized into four discrimination scenarios: (1) at an educational institution, (2) in the workplace, (3) in everyday life, (4) and at a healthcare facility. The data were collected via in-depth interviews. The outcomes should reflect the problem of discrimination against TGWs with an intersectional identity and related organizations and help stakeholders to provide plans to mitigate discrimination against them in the long run.

## 2. Materials and Methods

### 2.1. Participants

**Figure 1:**
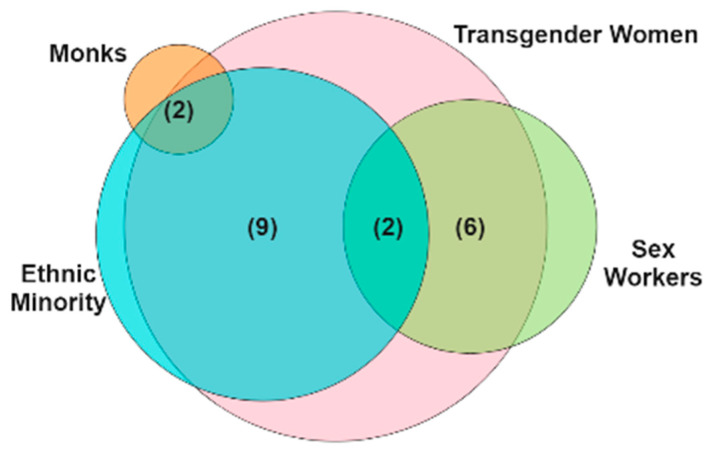
The number of participants with an intersectional identity (n).

The qualitative data used in this study were collected by the Mplus Foundation in Chiang Mai and the SISTERS foundation in Chonburi, Thailand, between 1 April 2022 and 31 May 2022. Nineteen participants aged 18 years old or older with intersectional identities, including (1) ethnic minority TGWs (9), (2) TGW sex workers (6), (3) ethnic minority TGW sex workers (2), and (4) ethnic minority TGW monks (2), were included in the study (Figure 1). Before beginning the interviews, the reasons for this study were explained to the participants, after which written consent was obtained from them.

### 2.2. Data Collection and Measurements

The interviews were carried out by an in-depth interview specialist (N.M.) who has been trained in counseling as well as mental health issues by the Mplus foundation. Since the data collection was conducted during the COVID-19 epidemic situation in Thailand, there were two methods of interviewing, in-person and by phone, which depended on what was convenient for each participant. When conducting the in-person interviews, the interviewer and the participant were in a private room, wearing masks, and staying more than 1 m apart for the entire process. This complied with the guidelines for preventing the spread of COVID-19 in Thailand. The schedule for the phone interview was arranged at the convenience of the participant and to ensure that the interviewer and participant were alone to maintain their privacy concerning personal information. The interviews with Buddhist priests were conducted over the phone and had to be formal, using religious terminology to ensure the security of the monk’s status while providing information.

The participants were interviewed in Thai for between 45 and 60 min. Interviews were digitally audio recorded and then transcribed verbatim, and pseudonyms were allocated to the interviewees to preserve their anonymity. The semi-structured interviews consisted of two parts: (1) demographic information, including age, ethnicity, education, and occupation, and (2) experience of perceived discrimination. Part 2 of the semi-structured interview was a modified version of the perceived ethnic discrimination questionnaire-community version (PEDQ-CV) by using the questions in the section concerning discrimination in the past among ethnic groups living in Asia [36,37].

### 2.3. Statistical Analysis

We categorized the data into four discrimination scenarios: (1) at an educational institution, (2) in the workplace, (3) in everyday life, (4) and at a healthcare facility. We rechecked the information and encoded the themes and sub-themes from the semi-structured in-depth interview transcripts using the NVivo program (release 1.3) prior to performing the statistical analysis. 

The descriptive statistics of the characteristics of the participants are presented as frequencies and percentages for categorical variables and medians and interquartile ranges for the age variable. Thematic analysis was used to identify patterns or themes from the interviews. A re-iterant process was used for discussing areas of agreement and disagreement.

### 2.4. Ethical Approval

This study was approved by the Chiang Mai University Research Ethic Committee (CMUREC 65/012). Informed consent was obtained from all of the subjects involved in the study.

## 3. Results

### 3.1. Participants

**Figure 2:**
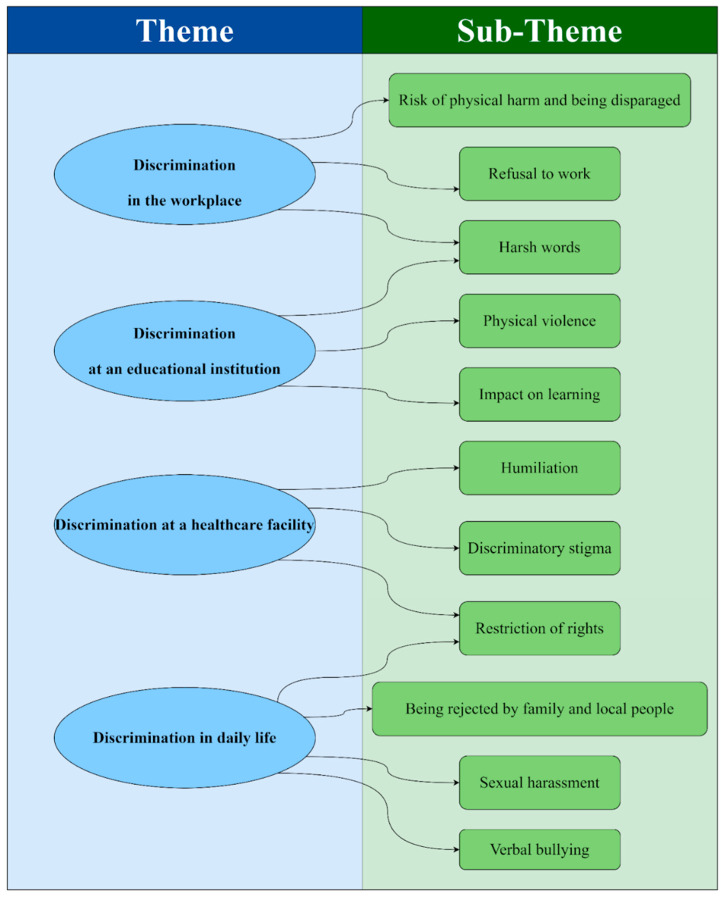
Themes and sub-themes used in the study.

The median age of the 19 participants was 30 years old (IQR = 28–40). The majority of the intersectional identities were ethnic minority TGWs (9/19; 47%), followed by TGW sex workers (6/19; 31%). Most of them were from the Thai Yai ethnic group (8/19; 42%), followed by Thai Yong and Thai Lue (21% and 5%, respectively). Most of them were self-employed as sex workers (8/19; 42%) and monks (2/19; 11%). Most of them had lived in Thailand for 11 years or more (7/19; 36%) (Table 1). The in-depth interviews were divided into four main themes with several sub-themes (Figure 2).

### 3.2. Discrimination at an Educational Institution

Discrimination was reported via the use of inappropriately harsh words by teachers, friends, and/or seniors at school because of having an intersectional identity, as per a TGW sex worker aged 30 years old. “Teachers tried to persuade me to transform back to being a man because society would not accept me, would bully me, and it would be difficult to find work.” (a TGW sex worker aged 27 years old); “I am from a rural community and my skin color is quite dark, and so I was often teased about being a black ladyboy.” (a TGW sex worker aged 29 years old); and “I was new to Thailand from Myanmar and couldn’t speak or write Thai, so I was often bullied for being “stupid”.” (a TGW monk from ethnic minority aged 29 years old) are some of the answers of the interviewees.

Some have been discriminated against through physical violence, and some are misunderstood and judged by their outward appearance by friends and teachers at educational establishments. For example, “A third-gender friend and I were often hit, kicked, teased, and caught naked by mischievous teenagers at school.” (a TGW sex worker aged 27 years old) and “A third-gender friend and I were often subjected to anger, violence, and being spoken rudely to.” (a TGW sex worker aged 26 years old).

Some have been impacted by both positive and negative discrimination at school. An example of a positive impact is “Being teased by a friend motivated me to study hard, and I received first place in the school.” (a TGW monk from ethnic minority aged 29 years old), and an example of a negative impact and unacceptable event is “The teacher told me that if they said it and I couldn’t accept it, then I should quit, and so I decided to stop studying and quit.” (a TGW sex worker aged 30 years old).

### 3.3. Discrimination in the Workplace

An example of workplace discrimination is people refusing to work with a group of individuals with intersectional identities. For example, “When I applied for a particular job, I was rejected because they wanted a woman due to the work being delicate, and when I applied to be a security guard, the company wouldn’t accept me because it required a man.” (a TGW from an ethnic minority aged 35 years old); “My female friend applied to be a hotel worker. The hotel needed more workers but when I went to apply for a job, I was not accepted because I’m a third-gender person.” (a TGW sex worker from an ethnic minority aged 28 years old); and “In addition, the organizational restrictions are another reason why LGBTQ people are denied employment. When I went to apply for a job, the interviewer said “when you come to work, you must dress as a man and talk less. If you act as a third-gender individual at work, you will not be accepted.” (a TGW from an ethnic minority aged 29 years old).

Harsh words or bullying remarks made by colleagues or even customers are other sources of discrimination at work. For example, “I’ve been accused of being homosexual and selling sex services, or some customers didn’t read my profile. When they saw me as a ladyboy, they rebuked me with harsh words.” (a TGW sex worker aged 27 years old).

In addition, sex workers are at risk of physical abuse and degradation. For instance, “Customers have sometimes argued and threatened bodily harm, and have not paid or paid only half the fee when they found out I wasn’t a woman.” (a TGW sex worker aged 28 years old), and “Some customers paid me less or had sex as a prank because they saw me as a transgender woman, which I can’t accept.” (a TGW sex worker aged 26 years old).

### 3.4. Discrimination in Daily Life

Sexist bullying words or mimicry can make someone feel despondent, especially when directed at particular minority groups. For instance, “I am often referred to as a homosexual. “ (a TGW sex worker aged 21 years old); “I’m often subjected to sexism, especially in Tai Yai society.” (a TGW from an ethnic minority aged 35 years old). Some of the participants have been humiliated by people they did not know. For example, “People looked at my dress and laughed and pointed at me. He called his friends to look at me and said loudly, “Is this a ladyboy or …?”.” (a TGW from an ethnic minority aged 29 years old).

Even though perpetrators think of it as a prank, it is actually a type of sexual harassment. For instance, “I was touched on my butt, chest, or genitals.” (a TGW from an ethnic minority aged 30 years old). 

During the Songkran festival, this group is often subjected to physical violence. For example, “I went to a Songkran festival with my friends when I was a teenager. People threw shoes and water bowls, and even placed a bucket of water on my head.” (a TGW sex worker aged 28 years old).

Being rejected by family and local people is another form of discrimination experienced in daily life. Some forms are related to religion and/or traditional beliefs. For instance, “People in the community say, “Why not be a man?”, “Why do you want to be a woman?”, or “It’s a sin”. It’s a sin that will fall on us and we will need to make merit to atone for it. They frequently comment about it even though the truth is not related.” (a TGW sex worker aged 27 years old), and “People in the community say they don’t like me and don’t want anything to do with me. They spoke specific words concerning hermaphrodites.” (a TGW from an ethnic minority aged 36 years old).

In addition, people with conflicting identities have restricted access, such as when they want to use public restrooms. This group of people often encounter problems in their daily lives, such as “When I need to go to the public restroom, I am always confused about whether I should go to the men’s or women’s. If I go to the women’s bathroom, the women will be angry and resentful. If I go to the men’s bathroom, men look at me.” (a TGW from an ethnic minority aged 52 years old); “Including rights that I should have access to but are restricted due to them only being available for Thai nationals and people with characteristics that match their birth gender only. This causes obstacles and restrictions for people with intersectional identities. For traveling outside the area or across the province, we need to apply for a permit for leaving the area at the district level, which causes inconvenience and hassle.” (a TGW from an ethnic minority aged 36 years old). Other examples are as follows: “My friend and I encountered difficulties with our name title prefix when traveling abroad. The interviewer asked us more questions than the others and often asked us if we are going to sell sex services?” (a TGW sex worker aged 26 years old). In addition, there are restrictions on marriage registration in Thailand that can make life as a couple difficult. For example, “My partner with whom I live in Thailand has questions about various lifestyles. There is no law to certify same-sex marriage, which makes living together here quite difficult. In his country, there is a law that allows same-sex marriage.” (a TGW sex worker aged 26 years old).

### 3.5. Discrimination at a Healthcare Facility

TGWs are often discriminated against at healthcare facilities because their name and title prefix do not match how they dress. For example, “I went to the hospital and was called Mr.... and I had to walk past other people who looked at me and laughed, embarrassing me like I was a clown, and so I didn’t want to receive healthcare services.” (a TGW from an ethnic minority aged 40 years old). Discrimination against TGW sex workers with intersectional identities, especially those working in brothels, is prevalent. They are asked to undergo additional health checks that are not related to their symptoms. When seeking treatment for a panic disorder, one participant was asked to consent to a blood test to check for thyroid functionality and questioned about taking hormones despite seeking treatment for an unrelated condition: “I went for treatment for a panic disorder. The additional blood test or unrelated health checks are just because I’m a transgender woman. This made my treatment cost much higher than for others.” (a TGW sex worker aged 26 years old).

Another important issue concerning discrimination at healthcare facilities is restricting the right to medical treatment. For instance, “I went to the hospital for treatment. I don’t have the right to medical care or personal health insurance, and so I had to pay all my expenses because I’m from an ethnic minority.” (a TGW from an ethnic minority aged 35 years old). When they want to help others by donating blood, their offer to help others is usually refused because they are LGBTQ. For example, “When I donated my blood, the staff saw me as a third-gender person and didn’t accept my blood even though I have no comorbid diseases.” (a TGW sex worker aged 30 years old).

### 3.6. The Effects of Intersectional Identities on Discrimination

Most TGW participants (12/19, 63.2%) have experienced discrimination related to refusing them employment at certain workplaces, followed by being rejected by their family and local people in their daily lives (9/19, 47.4%) and verbal bullying in their daily lives (7/19, 36.8%). Compared to the other intersectional identity groups, TGWs from ethnic minorities have experienced fewer forms of discrimination (1–5), whereas TGW sex workers have experienced more (3–7). Moreover, the number of discrimination forms is not related to the number of intersectional identities (Table 2).

## 4. Discussion

The findings from our study through in-depth interviews with 19 people revealed discrimination experienced by the intersectional identities group at school, at work, in daily life, and/or at a healthcare facility. The major discriminatory actions toward transgender people at school were insulting speech and/or even physical abuse. As well as being subjected to these, the intersectional identity groups had been insulted for belonging to an ethnic group, which had an impact on both positive and undesirable outcomes. The positive impact was that it encouraged them to study harder, whereas the negative impact forced them out of the school system. Likewise, in previous studies, these individuals are less likely than others to have a bachelor’s degree [1] and more likely to have poor mental health [38,39,40,41,42]. Raising awareness of gender diversity and intersectionality in schools and communities could be beneficial in reducing discrimination issues. Anti-bullying legislation should be enforced to prevent violence and bullying towards transgender people, especially at the school level.

We found no evidence to support that a higher number of intersectional identities affects the higher forms of discrimination against TGWs with intersectional identities. However, those in some categories of intersectional identity (such as TGW sex workers) experienced more forms of discrimination than others. Whether they belong or do not belong to an ethnic minority, most of them (6/8, 75%) reported that they had experienced discrimination when seeking employment. This might be one of the reasons that made them decide to become sex workers, even though they faced a higher risk of discrimination in their work and daily lives. This is consistent with the findings from a previous report in that the high amount of compensation and acceptance of their gender identity has enticed some TGWs in Thailand to enter the sex industry [21].

When considering employment discrimination, TGWs not only have limited access to employment but also experience conflict with colleagues and supervisors. Some employers require employees to dress according to their birth-assigned gender and also interfere with their chances of promotion [2]. For those who have chosen to become sex workers to support their families, degradation, physical abuse, and/or refusal to pay for services are quite common occurrences. Likewise, according to the outcomes of previous studies, these individuals often experience compounded discrimination and stigma that negatively affects the community [5,43]. As is generally known, although Thailand’s sex workers generate 6.4 billion dollars annually [44], prostitution is still illegal, immoral, unethical, and not accepted by Thai society, so there is no social security or healthcare available to them. Indeed, many have been physically attacked and arrested by government officers [4]. 

As an example of discrimination against individuals with an intersectional identity according to race, a research group from the US reported that 11.3% of LGBTQI employees of color experienced unfair treatment at work compared to 6.5% of white LGBTQI employees [20]. Anti-discrimination laws in employment against a job applicant or an employee on the grounds of race, gender expression, and gender identity would raise the employment opportunities for TGWs from an ethnic minority. Identifying individuals with an intersectional identity and comparing them with TGWs without one would enable us to comprehend differences in discrimination in the social contexts of family, school, workplace, and community. However, since TGWs without an intersectional identity were not included in the present study involving in-depth interviews, exploring these differences between the two groups cannot be provided herein.

Because they have been rejected by society and unappreciated by their families and communities, they often relocate from home to try and avoid discrimination. In addition, the main religion in Thailand is Buddhism, and most Buddhists believe that sin or karma is to blame for a person’s desire to have a different gender assignment from that given at birth [45]. This might have influenced how society perceives intersectional identity persons as different, thereby causing discrimination and difficulties with them integrating into society. Although there are still some people in Thailand who accept them and are working to mitigate the social stigma, changing the regulations to cater to third-gender people is not progressing quickly. Same-sex marriage and officially changing their gender assignment are unavailable, while labor laws only cater to cisgender individuals. These findings are consistent with those from previous studies [2,46]. 

Our findings suggest that intersectional people experience worse healthcare outcomes, which is supported by previous study outcomes. For instance, LGBTQ people in the UK have less favorable healthcare experiences than cisgender people [9]. One of the causes is that most healthcare professionals are not trained to deal with transgender patients, and as a result, the latter commonly encounter ambivalence and uncertainty when seeking medical intervention [31]. Consequently, discrimination occurs during subsequent interactions with caregivers [33]. A fundamental right should be the ability to access appropriate medical care and counseling for physical and mental wellbeing, especially for trans people. Therefore, in an effort to address discrimination and healthcare disparity, healthcare that is recognized as LGBTQ-friendly should be established, with expert care providers and convenient access. In addition, blood donation remains prohibited in Thailand for the LGBTQ community due to the perception that they are at greater risk of HIV or hepatitis B infection, even though everyone carries the same risk of contracting these infections. However, some countries accept blood donations for these groups under certain conditions, so they do not feel any different. For instance, in the US, blood donors registering as transgender are not deferred, and eligibility is based on the criteria associated with the gender the donor has reported [47]. However, in Australia, a man who has sex with men or who is a sex worker needs to wait 3 months before donating blood [48]. Raising the understanding, the quality of service, and the coverage of the healthcare providers in both physical and mental health is also important to improve the health outcomes and the healthcare service accessibility among the transgender women with intersectional identities.

As can be seen from our study outcomes, intersectional identity groups are stigmatized and discriminated against in every aspect of their lives with harsh words or sexual violence, at school or work, in everyday life or in healthcare settings. Even though school is supposed to be a safe and equitable environment free of discrimination for all students, it fosters violence and stigma against TGWs or minority students. Furthermore, some teachers aggravate rather than help the situation, causing some of them to drop out. The compulsory education level in Thailand is grade 9 in high school. Meanwhile, the job market is extremely competitive, and a good career is impossible to achieve without adequate education. Thai society only accepts transgender people conditionally, provided that they are beautiful, wealthy, and successful. At the other end of the spectrum, job security is extremely limited for anyone who engages in sex work. Their daily lives are difficult, and a lack of basic rights and sufficient healthcare prevents them from living normal lives. Moreover, a sex worker having an intersectional identity makes the situation even worse [49].

Although this is a small sample size interview with intersectional identities, the results of these in-depth interviews are extremely valuable since the participants with intersectional identities cannot be easily contacted. The study’s strength is that there has not been as much research published on intersectional identities as there is on transgender groups.

## 5. Conclusions

Although transgender identity is socially acceptable in Thailand to a certain degree, stigma and discrimination are still important issues, especially for transgender women with an intersectional identity. The outcomes of this qualitative in-depth interview study reflect the problems associated with multiple sources of discrimination aimed at transgender women with an intersectional identity in Thailand, including harsh speech or physical abuse; occupational, social, and legal inequality; and healthcare provision disparities. Raising awareness about gender diversity and intersectionality, as well as enforcing anti-bullying legislation and anti-discrimination laws, should be continually pursued in order to protect the rights and improve the quality of life of transgender individuals with an intersectional identity. In addition, enabling the rights of transgender people by allowing changes in gender assignment and same-sex marriages, as well as providing better welfare, will reduce the inequality between the cisgender and transgender populations.

## Figures and Tables

**Table 1:** The characteristics of the participants.

Characteristic	n	Percent
Age (years old)		
Median of age (interquartile range)	30 (28–40)	
Intersectional nature		
Ethnic minority TGW	9	47.0
TGW freelancer	6	31.0
Ethnic minority TGW freelancer	2	11.0
Ethnic minority TGW monk	2	11.0
Ethnicity		
Tai Yong	4	21.0
Tai Lue	1	5.0
Tai Yai	8	42.0
Thai	6	32.0
Status		
Single/widowed	8	42.0
Married/have a boyfriend or girlfriend	3	16.0
Not specified	8	42.0
Education level		
Uneducated/primary school	4	21.0
Secondary school/vocational certificate	10	53.0
Higher vocational certificate/bachelor’s degree/postgraduate	5	26.0
Occupation		
Government employee/private business owner	7	36.0
Monk	2	11.0
Freelancer	8	42.0
Unemployed	2	11.0
Sex worker status		
Yes	8	42.0
No	11	58.0
Length of stay in Thailand		
Since birth	6	32.0
1–10 years	1	5.0
≥11 years	7	36.0
Not specified	5	27.0

**Table 2:** Intersectional identities and multiple discriminatory actions.

		ID																		
Situation	Type of Discrimination	1	2	3	4	5	7	10	16	19	11	13	14	15	17	18	6	12	8	9
Workplace	Physical harm, being disparaged											√	√	√	√					
	Refusal of employment		√	√		√		√	√	√	√	√	√		√	√		√		
	Harsh words											√		√		√	√	√		
Educational Institution	Hash words										√	√	√			√			√	√
	Physical violence							√				√	√			√				
	Impact on learning																		√	√
Healthcare	Humiliation									√										
	Discrimination and stigma							√					√							
	Restriction of rights		√									√			√	√				√
Daily life	Restriction of rights		√						√				√							
	Being rejected by family and local people	√	√	√			√	√		√				√		√				√
	Sexual harassment									√									√	
	Verbal bullying		√		√			√	√		√	√						√		
Number of discrimination forms		1	5	2	1	1	1	5	3	4	3	7	6	3	3	6	1	3	3	4
Number of intersectional identities		2	2	2	2	2	2	2	2	2	2	2	2	2	2	2	3	3	3	3
Intersectional identity categories		C1									C2						C3		C4	

C1, TGWs from an ethnic minority; C2, TGW sex workers; C3, TGW sex workers from an ethnic minority; C4, TGW monks from an ethnic minority. The "√" sign was represented for the type of discrimination that the responses had experienced.

## Data Availability

Not applicable.
